# Lung Involvement Found on Chest CT Scan in a Pre-Symptomatic Person with SARS-CoV-2 Infection: A Case Report

**DOI:** 10.3390/tropicalmed5020056

**Published:** 2020-04-07

**Authors:** Ali Asadollahi-Amin, Mehrdad Hasibi, Fatemeh Ghadimi, Hosnieh Rezaei, SeyedAhmad SeyedAlinaghi

**Affiliations:** 1 Iranian Research Center for HIV/AIDS, Iranian Institute for Reduction of High-Risk Behaviors, Tehran University of Medical Sciences, Tehran 1419733141, Iran; amiyaneh@gmail.com (A.A.-A.); fatemeh.ghadimi@gmail.com (F.G.); 2 Department of Infectious Diseases, Amir Alam Hospital, Tehran University of Medical Sciences, Tehran 131851678, Iran; mehrdad_hasibi@yahoo.com (M.H.); hosniehrezaei@yahoo.com (H.R.)

**Keywords:** COVID-19, SARS-CoV-2, pneumonia, emerging infectious diseases

## Abstract

The novel coronavirus SARS-CoV-2 infection is spreading worldwide, and there are many reports of acute respiratory distress syndrome caused by this infection. However, asymptomatic lung involvement has not been reported. We hereby present the case of a 44-year-old health-care worker, who was found to be infected with the SARS-CoV-2 virus after a CT-scan performed for an unrelated condition revealed a lesion in the lung field compatible with COVID-19 infection. His condition deteriorated initially, but eventually improved with supportive treatment and the compassionate use of antivirals and antimalarials and is now in a stable condition.

## 1. Introduction

The outbreak of coronavirus disease 2019 (COVID-19) began in Wuhan, China, in December 2019, and since then, it has spread throughout the world [1,2].

The severe acute respiratory syndrome coronavirus 2 (SARS-CoV-2) responsible for COVID-19 causes a spectrum of illness, from asymptomatic infections to pneumonia, and in severe cases respiratory failure, shock, or multiorgan dysfunction [3].

Here, we present a case of an individual infected with SARS-CoV-2 whose apparent lung involvement on chest CT scan was found accidentally. This case highlights the possibility of severe lung involvement with this emerging infection while the individual is still symptom-free.

## 2. Case Report

A 44-year-old male health care worker presented to the emergency department of a hospital in Tehran, Iran. The hospital was a designated coronavirus referral center at the time. He described a history of rib fracture due to falling about two weeks ago and complained of local tenderness and pain that was not responsive to over-the-counter painkillers. Upon admission to the emergency room, vital signs were within normal ranges. He reported no underlying medical conditions, and the patient was otherwise healthy.

**Figure 1:**
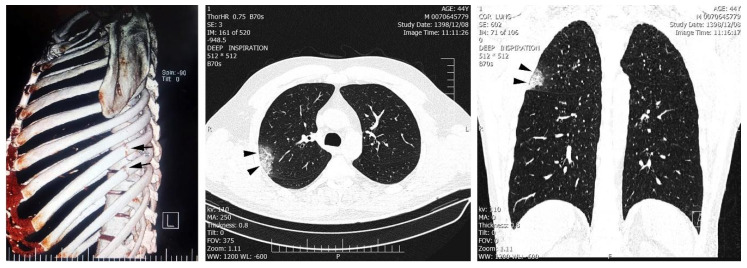
Fractures are seen in the left 8th and 9th ribs (arrow). Peripheral patchy ground-glass opacity in the right upper lobe is observed due to viral pneumonia (arrowheads). Neither pleural effusion nor lymphadenopathy were found. Written informed consent was obtained from the patient for publication of this case report.

A chest CT scan was performed (Figure 1), which revealed left 8th and 9th ribs fracture along with an ill-defined patchy ground-glass opacity in the upper lobe of the right lung. Due to high suspicion of SARS-CoV-2 infection, an upper respiratory tract swab for severe acute respiratory syndrome coronavirus-2 (SARS-CoV-2) was obtained, and a positive real-time reverse-transcriptase polymerase chain reaction (rRT-PCR) assay confirmed the diagnosis of COVID-19 infection.

Based on the Iranian interim guideline for "clinical management of COVID-19" published in February 2020, the decision for the use of antiviral and antimalarial therapy was made considering the patient’s diagnosis of SARS-CoV-2 infection [4]. Oseltamivir 75 mg every 12 hours, and hydroxychloroquine 400 mg stat was initiated despite no clinical signs. After three days of the treatment, fever and dyspnea developed, and the patient’s oxygen saturation values dropped to 93% while he was breathing room air.

The treatment regimen was changed to a more focused antiviral therapy with oseltamivir 75 mg and lopinavir/ritonavir (Kaletra), 400/100 mg every 12 h [4]. After approximately 24 h, the patient’s clinical condition started to improve; fever abated, dyspnea decreased, and O_2_ saturation increased to 97% on room air. On the fifth day of admission, the patient became asymptomatic.

## 3. Discussion

Although knowledge of COVID-19 is evolving, there is still limited data available around the transmission dynamics and the full range of clinical spectrum of SARS-CoV-2 infection. Most of the currently available data are based on the cases identified with a pneumonia diagnosis. Although asymptomatic infections have also been described, the data around the individuals in incubation period is lacking [5,6,7].

Here we reported the case of a symptom-free individual accidentally found to be infected with SARS-CoV-2, which illustrates a new aspect of this newly emerging infection. Despite significant radiographic abnormalities, our patient experienced a milder form of pneumonia and did not require intubation or supplemental oxygen.

Detection of SARS-CoV-2 infection at early stages would allow for immediate treatment of the patient and possibly lower the adverse outcomes. Additionally, early identification of the cases leads to prompt isolation and subsequently reduces transmission, slowing the potential spread. Both highlight the need for a rapid, sensitive diagnostic tool that allows managing patients and controlling outbreaks more efficiently. In the absence of such a tool, high clinical suspicion in the epidemic area helps timely detection of the patients to ensure early isolation and treatment.
